# A neural network model for predicting the effectiveness of treatment in patients with neovascular glaucoma associated with diabetes mellitus

**DOI:** 10.22336/rjo.2024.53

**Published:** 2024

**Authors:** Olga Volodymyrivna Guzun, Oleg Serhiyovich Zadorozhnyy, Volodymyr Viktorovych Vychuzhanin, Natalia Ivanivna Khramenko, Liudmyla Mykolayivna Velichko, Andrii Rostyslavovich Korol, Valeriu Nicon Cușnir, Lilia Gheorghe Dumbrăveanu, Vitalie Valeriu Cușnir

**Affiliations:** 1 “The Filatov Institute of Eye Diseases and Tissue Therapy of the National Academy of Medical Sciences of Ukraine” State Institution, Odesa, Ukraine,; 2 “Odesa Polytechnic” National University, Odesa, Ukraine,; 3 Department of Ophthalmology and Clinical Optometry, “Nicolae Testemițanu” State University of Medicine and Pharmacy, Chişinău, Republic of Moldova

**Keywords:** neural networks, diabetic neovascular glaucoma, systemic inflammation, intraocular microcirculation, AI = artificial intelligence, T2DM = type 2 diabetes mellitus, DR = diabetic retinopathy, PDR = proliferative diabetic retinopathy, NVG = neovascular glaucoma, TSC CPC = transscleral cyclophotocoagulation, IOP = intraocular pressure, ROG = rheoophthalmography, RQ = rheographic coefficient, SII = systemic immune-inflammation index, SIRI = systemic inflammation response index

## Abstract

**Introduction:**

The study hypothesizes that neural networks can be an effective tool for predicting treatment outcomes in patients with diabetic neovascular glaucoma (NVG), considering not only baseline intraocular pressure (IOP) values but also inflammation and intraocular microcirculation indicators.

**Objective:**

To investigate the diagnostic significance of inflammation and intraocular blood circulation indicators in a neural network model predicting the effectiveness of transscleral cyclophotocoagulation (TSC CPC) treatment in patients with NVG of diabetic origin.

**Methods:**

This retrospective cohort study included 127 patients (127 eyes; aged Me 65.0 years) with painful diabetic NVG and 20 healthy individuals (aged Me 61.5 years) as an immunological control. All patients underwent TSC CPC with a diode laser. Treatment success was defined as achieving an IOP level of ≤ 21 mmHg and maintaining or improving best-corrected visual acuity (BCVA) after 12 months of observation. Preoperative systemic immune-inflammation index (SII = platelets × [neutrophils/lymphocytes]) and systemic inflammation response index (SIRI = neutrophils × [monocytes/lymphocytes]) were calculated. We assessed the values of volumetric pulse blood filling, determined by the rheographic coefficient (RQ, 0/00), using the rheoophthalmography (ROG) method. Multiple regression analysis was used to conclude the significance of treatment efficacy based on initial clinical and laboratory indicators, followed by constructing a prediction model in the neural network.

**Results:**

The development of the neural network model identified the most significant “input” parameters: SIRI (100%), RQ (85.7%), and SII (80.7%), which significantly influenced treatment success. The sensitivity of the neural network model was 100%, specificity was 30%, and the percentage of correctly predicted events during testing on the control group was 92.9%.

**Conclusions:**

Neural network-based prediction of transscleral cyclophotocoagulation effectiveness for patients with diabetic neovascular glaucoma allows for a sufficiently accurate forecast of treatment success with a probability of 92.9%. We believe the in-time correction of systemic inflammation and intraocular blood circulation can significantly reduce intraocular pressure, preserve visual acuity, and improve the quality of life in patients with diabetic NVG after TSC CPC. Further research is required to support these findings.

## Introduction

Currently, about 1% of the blind or visually impaired population worldwide is due to proliferative diabetic retinopathy (PDR), which is one of the leading causes of secondary neovascular glaucoma (NVG). NVG is characterized by rubeosis iridis, the formation of a fibrovascular membrane in the angle of the anterior chamber, accompanied by high intraocular pressure (IOP) and often severe ocular pain [1].

The growing prevalence of type 2 diabetes mellitus (T2DM) has been noted in the epidemiological study by Kiro et al. amidst the stress caused by the war in Ukraine. The authors recorded a significant increase in average fasting glucose levels and glycated hemoglobin (HbA1c) [2].

The hypothesis of subclinical inflammation in T2DM, influencing the development of PDR is considered by many authors. Damaged microvasculature, endothelial dysfunction with increased vascular permeability, retinal ischemia, and subsequent neovascularization contribute to neuroinflammation and neurodegeneration. In patients with progressing diabetic retinopathy (DR), levels of inflammatory mediators increase, as does the number of adhesion molecules (ICAM-1 and VCAM-1) [3-5].

Leukocytes accumulate at the injury site during inflammation, and the local blood flow rate decreases. Microcirculation disorders in the retina are observed in patients with T2DM without clinically evident diabetic retinopathy [6]. Wang et al. analyzed global studies on diabetic kidney disease over 32 years and noted therapeutic strategies aimed at improving renal microcirculation in these patients [7]. Changes in blood flow in T2DM patients contribute to macrovascular (peripheral vascular disease and coronary artery disease) and microvascular (diabetic retinopathy and diabetic nephropathy) complications. Li et al. found significant associations between retinal microcirculation (vessel density and perfusion density in the superficial capillary plexus and foveal avascular zone) with lipid profile, kidney function, and platelets in patients with T2DM [8]. Vascular endothelial damage increases adhesion factors, enhancing coagulation and leukocyte adhesion, thus, leading to a decrease in blood flow rate.

To relieve pain and reduce IOP in patients with secondary NVG of diabetic origin, transscleral (TSC) cyclophotocoagulation (CPC) is widely used. The therapeutic mechanism of this method involves reducing the production of intraocular fluid by the ciliary body. According to the literature, CPC achieves an 88.6% success rate in treating glaucoma patients [9]. However, in some patients, IOP reduction is insufficient, and they require repeated treatment courses. These patients exhibit inflammatory processes (elevated levels of neuroinflammatory proteins [10]) and microvascular disorders in the eye. Surgical interventions are often ineffective for this patient category due to elevated cytokine levels [11].

Today, automated tools for screening, monitoring, and predicting eye diseases are being developed actively. Ahn et al. (2018) built a deep-learning model for diagnosing glaucoma using 1,542 fundus photographs (786 healthy individuals, 467 confirmed glaucoma cases with signs of progression, and 289 photos of patients with early-stage glaucoma) [12]. They used these datasets to build a simple logistic classification and a convolutional neural network. Additionally, based on fundus images, Kim et al. (2017) and Raghavendra et al. (2018) developed neural networks capable of diagnosing glaucoma in patients, equivalent to an expert consultant [13]. Li et al. (2018) proposed a deep learning classification system for glaucomatous optic neuropathy (GON), retrospectively studying 48,116 color fundus photographs [14]. Some authors use OCT data to implement deep learning algorithms and quantitatively assess glaucomatous structural damage [15,16]. Artificial intelligence (AI) provides a more effective and accurate interpretation of numerous patient data sets [17]. AI can be implemented using several approaches, including machine learning and deep learning with convolutional neural networks.

Deep learning involves iterative algorithms and system self-correction by adjusting algorithm parameters to reduce diagnostic errors. It is already being used to screen for diabetic retinopathy by interpreting color fundus photographs of patients with diabetes [18].

Thus, using neural networks can be an effective tool for predicting treatment outcomes in patients with diabetic NVG, considering not only baseline IOP values but also inflammation and intraocular microcirculation indicators.

The study aimed to investigate the diagnostic significance of inflammation and intraocular blood circulation indicators in a neural network model predicting the effectiveness of transscleral cyclophotocoagulation treatment in patients with neovascular glaucoma of diabetic origin.

## Materials and methods

### Study Population

A retrospective cohort study was conducted at “The Filatov Institute of Eye Diseases and Tissue Therapy of the National Academy of Medical Sciences of Ukraine” State Institution. The study protocol adhered to the principles of the Helsinki Declaration and was approved by the institution’s local bioethics committee. Written informed consent was obtained from all study participants.

The study participants included both outpatient and inpatient patients with T2DM suffering from painful neovascular glaucoma. For controlling immunological characteristics, data from 20 healthy individuals were used to determine the expression of intercellular adhesion molecule-1 (ICAM-1) on peripheral blood lymphocytes (CD54 marker) (**Table 1**).

**Table 1:** Demographic and clinical characteristics of patients with neovascular glaucoma of diabetic origin

Characteristic	Value Me (Q 25%-75%) or n (%)
Age (years), n=127	65,0 (62-68)
Control, n=20	61,5 (56,5-65)
Men/Women	60 (47%)/67 (53%)
Intraocular Pressure (IOP, mmHg)	34,0 (33-39)
Number of Anti-Glaucoma Medications	3,0 (2-3)
Anti-Glaucoma Interventions	31 (24%)
Cataract/Pseudophakia	76 (60%)/51 (40%)
BCVA < 0.02	34 (27%)
BCVA ≥ 0.02	93 (73%)
Retinal Photocoagulation	
Yes/No	42 (33%)/85 (67%)
Anti-VEGF Therapy	
Yes/No	26 (20%)/101 (80%)
Ophthalmoscopic Findings:	
-Corneal Dystrophy	21 (16,5%)
-Corneal Edema	27 (21%)
-Retinal Detachment	42 (33%)
-Epiretinal Membrane	33 (26%)
-Hyphema/Hemorrhage	18 (14%)/15 (12%)
Duration of Diabetes (years)	11,0 (7-14)
HbA1c (%)	7,4 (6,9-9,0)
Platelets (×10^9/L)	197 (182; 244)
Neutrophils (×10^9/L)	4,0 (3,8; 4,2)
Lymphocytes (×10^9/L)	1,9 (1,7; 1,9)
Monocytes (×10^9/L)	0,26 (0,26; 0,42)
SIRI (×10^9/L)	0,62 (0,52; 1,07)
SII (×10^9/L)	424,4 (383,8; 651,9)
CD54 Expression on Peripheral Blood Lymphocytes,	
-Absolute Value (cells/μL)	
-Relative Value (%)	580,0 (345,0-566) /
Control, n=20	26,0 (24-33)
-Absolute Value (cells/μL)/	
-Relative Value (%)	216,0 (187-234) /
	14,0 (12-17)
RQ, 0/00 (examined eye)	3,2 (2,6; 3,6)
RQ, 0/00 (the other eye)	3,4 (2,9; 3,7)
Control, n=20	3,3 (3,2; 3,5)
Smoking	28 (22%)

Note: Data are presented as median (Me), interquartile range (Q25%-Q75%), absolute numbers (n), and percentages (%). Abbreviations: IOP = Intraocular Pressure, BCVA = Best-Corrected Visual Acuity, PRP = Panretinal Photocoagulation, HbA1c = Glycated Hemoglobin, SII = Systemic Immune Inflammation Index, SIRI = Systemic Inflammation Response Index, RQ = Volumetric Index of Intraocular Blood Flow as measured by ROG.

Inclusion Criteria: 1. Diagnosed with neovascular glaucoma of diabetic origin; 2. Uncontrolled intraocular pressure (IOP) ≥ 30 mmHg; 3. Presence of ocular pain.

Exclusion Criteria: 1. Secondary NVG of non-diabetic origin; 2. Severe general somatic pathology that precluded performing TSC CPC; 3. Absence of pain syndrome.

Treatment Success Criteria: Achieving an IOP level ≤ 21 mmHg and maintaining or improving best-corrected visual acuity (BCVA) after 12 months of follow-up.

Study Visits: A preoperative visit (V0) was conducted the day before the laser procedure.

Subsequent visits were scheduled at 1 month (V1), 3 months (V3), 6 months (V6), and 12 months (V12). Treatment efficacy was analyzed after 12 months (V12). During these visits, BCVA, IOP (measured with a Goldmann applanation tonometer), burden of anti-glaucoma medications, presence of pain syndrome, and clinical signs of inflammation were assessed. Biomicroscopy, ophthalmoscopy, and gonioscopy were performed.

Preoperative laboratory parameters included neutrophils, lymphocytes, platelets, and monocytes. Systemic immune-inflammation index (SII = platelets × [neutrophils/lymphocytes]) and systemic inflammation response index (SIRI = neutrophils × [monocytes/lymphocytes]) were calculated. CD54 molecule expression on peripheral blood lymphocytes was determined using the histoimmunocytochemical method.

Regional hemodynamics was assessed using the rheoophthalmography (ROG) method and was performed on a computerized rheographic complex (Reocom, Ukraine). We assessed the volumetric pulse blood-filling parameters determined by the rheographic coefficient (RQ, 0/00) [19].

TSC CPC Procedure: Diode laser with a wavelength of 810 nm was used, following standard techniques. Selective energy parameters of laser radiation were used (median 36 (29.4-41.4) Joules per session): power ranged from 850 to 1500 mW (median 1100 mW), the exposure time was 1.5-2 seconds, and the average number of laser coagulates was 22 [5]. During each postoperative visit, the necessity for repeated TSC CPC was determined, with the criterion being the failure to achieve treatment efficacy one month after the laser intervention. The regimen of local hypotensive drugs was determined individually at each visit.

### Statistical Analysis

The data were processed using the Statistica software (version 10.0, StatSoft Inc., USA). The normality of continuous data distribution was checked using the Shapiro-Wilk test. Results were expressed as numbers and percentages (%) using frequency tables. Data are presented as the mean (M) and standard deviation (S), as well as the median (Me) and interquartile range (Q25%-Q75%).

A pairwise correlation of two independent samples was performed to compare multiple independent samples, using the Mann-Whitney U test, and the Kruskal-Wallis test was used to check the equality of medians among several samples. For repeated within-group comparisons, the Wilcoxon signed-rank test was applied (comparing V0 and V12). Spearman’s rank correlation coefficient (rs) was used to analyze the relationship between variables. A critical significance level of p<0.05 was adopted for testing statistical hypotheses.

Multiple regression analysis was used to determine the significance of treatment efficacy based on initial clinical and laboratory indicators, followed by constructing a prediction model in the neural network.

## Results

A total of 127 patients (127 eyes) with painful NVG against the background of PDR were examined and treated. The average age of the patients was 65.0 years (range: 62-68). The initial demographic and clinical characteristics of the patients recorded at the preoperative visit (V0) are presented in **Table 1**.

The development of the neural network prediction model was carried out in two stages. The first stage involved the selection of input parameters and their relationship with the outcome parameter (treatment success) through correlation and regression analysis (**Tables 2, 3**). The second stage involved the actual construction of the neural network model.

**Table 2:** Spearman’s rank correlation coefficient (r) of treatment success with investigated input parameters

Parameter	HbA1c	Diabetes Duration	IOP	CD54	SII	SIRI	RQ
Treatment Success	-0,79	-0,59	-0,63	-0,86	-0,79	-0,80	0,27

**Table 3:** Regression results for the dependent variable (treatment success) based on input parameters

Adjusted R^2^= 0,81; F (3,95) = 137,46; p=0,000; Std. Error of estimate: 8,084						
	Beta	Std. Err. of Beta	β	Std. Err. of β	t (83)	p-level
Intercept			1,669	0,162	10,310	0,000
SII	-0,491	0,097	-0,002	0,000	-5,050	0,000
SIRI quartiles	-0,259	0,100	-0,111	0,043	-2,594	0,011
RQ quintiles	0,206	0,073	0,205	0,072	2,837	0,006

Note: Beta: The regression coefficient shows the change in the dependent variable (treatment success) for a one-unit change in the predictor variable, holding all other variables constant; β (Standardized Beta): The standardized regression coefficient that indicates the strength and direction of the relationship between each predictor and the dependent variable, measured in standard deviations; Adjusted R^2^: The coefficient of determination adjusted for the number of predictors in the model; F: The calculated F-statistic for the overall significance of the regression model; df: The degrees of freedom associated with the F-statistic; p: The p-value for the F-statistic; Standard Error of Estimate: The average distance observed values fall from the regression line; Intercept: The value of the dependent variable (treatment success); Std. Error: The standard error of the intercept; t: The t-statistic for the intercept or each regression coefficient; p (for Intercept): The p-value for the t-statistic of the intercept

The correlation-regression analysis revealed a strong relationship between the treatment success (where 1 indicated an improvement (treatment success), and 0 - no improvement) and the input factors, specifically the number of patients exhibiting these changes.

From the sample observations (n=127), 83 patients (65%) experienced a reduction in IOP to ≤ 21 mmHg and had a stable or improved BCVA (1), whereas 44 patients (35%) had insufficient IOP reduction (remained > 22 mmHg) and experienced a decrease in BCVA (0). A multiple regression analysis was performed, which identified a strong correlation coefficient (**Table 3**).

Based on the correlation-regression analysis, the following parameters were included in the neural network model: SIRI quartiles, SII, and RQ quintiles. The outcome parameter for the neural network was treatment success (1). The architecture of the multilayer perceptron was selected automatically using Statistica 10, with a minimum of 1 neuron and a maximum of 50 neurons in the hidden layer. The neural network was constructed using a multilayer perceptron (**Table 4**).

**Table 4:** Results of the model for the dependent variable (treatment success) based on input parameters (SIRI quartiles, SII, and RQ quintiles)

Model Architecture	MLP 7-34-2
Training Set Performance	95,77465
Validation Set Performance	92,85714
Test Set Performance	100,0000
Training Algorithm	BFGS 1

### Model Formation and Evaluation

Three datasets were used to develop the perceptron model: Training Set: 70% of the data; Test Set: 15% of the data; Validation Set: 15%.

**Fig. 1:**
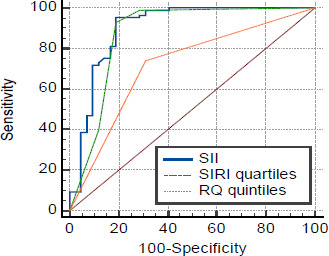
The ROC curve for the neural network’s input parameters is shown with the following values: SIRI: AUC = 0.953 ± 0.025; Sensitivity = 100%; Specificity = 88.64%; SII Quartiles: AUC = 0.921 ± 0.029; Sensitivity = 80.7%; Specificity = 90.9%; RQ Quintiles: AUC = 0.860 ± 0.036; Sensitivity = 85.7%

The output of the treatment effectiveness prediction included Neural Network Structure (description, diagram, and neuron weights), Network Performance (summary of the model, classification results, ROC curve (**Fig. 1**), cumulative diagram, gain chart, precision chart, and a summary report of observations), Analysis of Input Parameters (importance of input parameters used in the neural network).

Because of the neural network training analysis, the most optimal multilayer perceptron model was obtained. The sensitivity of the neural network model was 100%, the specificity was 30%, and the percentage of correctly predicted events during testing on the control group was 92.9% (**Table 5**).

**Table 5:** Summary data of the Neural Network Model (5.MLP 7-34-2)

	Predicted Values (%)		Treatment Effectiveness (0)	Treatment Effectiveness (1)	Treatment Effectiveness(all)
1		Training Sample			
	Correct		91,2	100	95,8
	Incorrect		8,8	0	4,2
2		Test Sample			
	Correct		100	100	100
	Incorrect		0	0	0
3		Control Sample			
	Correct		66,7	100	92,9
	Incorrect		33,3	0	7,1

## Discussion

Secondary NVG in patients with diabetes mellitus is a severe type of secondary glaucoma that is most resistant to both medical and surgical treatment, with the onset of pain making it an acute problem. NVG significantly impairs the quality of everyday life and can lead to complete blindness in these patients. Therefore, timely diagnosis and methods for predicting the effectiveness of treatments are crucial. Computer technologies are increasingly used in medical decision-making, and significant advances have recently been achieved in ophthalmology through artificial intelligence (AI) methods [20,21]. AI can successfully analyze and classify data from visual fields, optic nerve structures (primarily data obtained from OCT and fundus photography), and biomechanical properties of the eye. This helps determine the severity of the disease, its progression, and/or the need for specialized treatment.

Some researchers consider various machine learning and deep learning methods for successfully developing AI models in glaucoma, using fundus images and visual fields to detect glaucoma, assess its progression, and stage the disease [20]. Other authors have applied multimodal generative AI systems, such as the Chat Generative Pretrained Transformer (ChatGPT), for glaucoma diagnosis based on clinical reports. This approach has shown comparative effectiveness with the diagnosis made by experienced ophthalmologists [21]. However, colleagues from Japan emphasize the need for further development of multimodal generative AI systems for effective integration and use of clinical imaging data [22].

All these studies have focused on the diagnostic aspects of the disease [23]. In our study, we demonstrated high accuracy in predicting the effectiveness of TSC CPC in painful neovascular glaucoma of diabetic origin over 12 months using a neural network model. The model achieved a high accuracy in predicting treatment effectiveness with a probability of 92.9%. Accurate methods for identifying patients at high risk of potential complications are crucial when discussing treatment strategies. Our results are corroborated by Okruszko et al., who found elevated levels of neuroinflammatory proteins in patients with glaucoma, similar to those in neurodegenerative and autoimmune diseases [10]. Therefore, early detection of inflammatory and microcirculatory changes in patients can aid in the timely correction of these disturbances and in deciding on surgical or laser interventions, potentially reducing the risk of complications and preserving vision and quality of life for these patients.

Our results confirm that higher values of SIRI and SII indices, as well as RQ, indicate the severity of the disease and necessitate additional anti-inflammatory and anti-ischemic treatment for patients with painful NVG secondary to PDR to improve treatment outcomes. Given the significant inflammatory process, and disrupted intraocular volumetric blood circulation in these patients, a negative surgical prognosis can be anticipated [11]. Therefore, we suggest considering elevated levels of these indices and incorporating them into the absolute indicators for TSC CPC in patients with painful diabetic NVG to prevent surgical complications.

## Conclusion

Neural network-based prediction of transscleral cyclophotocoagulation effectiveness for patients with diabetic neovascular glaucoma allows for a sufficiently accurate forecast of treatment success with a probability of 92.9%.

The development of the neural network model identified the most significant “input” parameters: SIRI (100%), RQ (85.7%), and SII (80.7%), which significantly influenced treatment success.

We believe that timely correction of intraocular blood volume (RQ), the systemic inflammatory response index (SIRI), and the systemic inflammation index (SII) can significantly reduce intraocular pressure, preserve visual acuity, and improve the quality of life in patients with diabetic neovascular glaucoma after transscleral cyclophotocoagulation. Further research is required to support these findings.

## References

[1] Tang Y, Shi Y, Fan Z (2023). The mechanism and therapeutic strategies for neovascular glaucoma secondary to diabetic retinopathy. Front Endocrinol (Lausanne).

[2] Kiro L, Zak M, Chernyshov O (2023). Structure and dynamics of the course of chronic non-infectious somatic diseases in patients during war events on the territory of Ukraine. BMC Public Health.

[3] Siddiqui K, George TP, Mujammami M, Isnani A, Alfadda AA (2023). The association of cell adhesion molecules and selectins (VCAM-1, ICAM-1, E-selectin, L-selectin, and P-selectin) with microvascular complications in patients with type 2 diabetes: A follow-up study. Front Endocrinol (Lausanne).

[4] Blum A, Pastukh N, Socea D, Jabaly H (2018). Levels of adhesion molecules in peripheral blood correlate with stages of diabetic retinopathy and may serve as bio markers for microvascular complications. Cytokine.

[5] Guzun OV, Zadorozhnyy OS, Velychko LM, Bogdanova OV, Dumbrăveanu LG, Cuşnir VV, Korol AR (2024). The effect of the intercellular adhesion molecule-1 and glycated haemoglobin on the management of diabetic neovascular glaucoma. Rom J Ophthalmol.

[6] Cankurtaran V, Inanc M, Tekin K, Turgut F (2020). Retinal Microcirculation in Predicting Diabetic Nephropathy in Type 2 Diabetic Patients without Retinopathy. Ophthalmologica.

[7] Wang B, Xu M, Fu S, Wang Y, Ling H, Li Y, Li B, Liu X, Ouyang Q, Zhang X, Li A, Zhang X, Liu M (2024). Tiny clue reveals the general trend: a bibliometric and visualized analysis of renal microcirculation. Ren Fail.

[8] Li Y, Wu K, Chen Z, Xu G, Wang D, Wang J, Bulloch G, Borchert G, Fan H (2023). The association between retinal microvasculature derived from optical coherence tomography angiography and systemic factors in type 2 diabetics. Front Med (Lausanne).

[9] Bernardi E, Töteberg-Harms M (2022). MicroPulse Transscleral Laser Therapy Demonstrates Similar Efficacy with a Superior and More Favorable Safety Profile Compared to Continuous-Wave Transscleral Cyclophotocoagulation. J Ophthalmol.

[10] Okruszko MA, Szabłowski M, Zarzecki M, Michnowska-Kobylińska M, Lisowski Ł (2024). Inflammation and Neurodegeneration in Glaucoma: Isolated Eye Disease or a Part of a Systemic Disorder?-Serum Proteomic Analysis. J Inflamm Res.

[11] Chono I, Miyazaki D, Miyake H, Komatsu N, Ehara F, Nagase D, Kawamoto Y, Shimizu Y, Ideta R, Inoue Y (2018). High interleukin-8 level in aqueous humor is associated with poor prognosis in eyes with open angle glaucoma and neovascular glaucoma. Sci Rep.

[12] Ahn JM, Kim S, Ahn K-S (2018). A deep learning model for the detection of both advanced and early glaucoma using fundus photography. PLoS ONE.

[13] Raghavendra U, Fujita H, Bhandary SV (2018). Deep convolution neural network for accurate diagnosis of glaucoma using digital fundus images. Information Sciences.

[14] Li Z, He Y, Keel S (2018). Efficacy of a deep learning system for detecting glaucomatous optic neuropathy based on color fundus photographs. Ophthalmology.

[15] Medeiros FA, Jammal AA, Thompson AC (2019). From machine to machine: an OCT-trained deep learning algorithm for objective quantification of glaucomatous damage in fundus photographs. Ophthalmology.

[16] Rasel RK, Wu F, Chiariglione M, Choi SS, Doble N, Gao XR (2024). Assessing the efficacy of 2D and 3D CNN algorithms in OCT-based glaucoma detection. Sci Rep.

[17] Haja SA, Mahadevappa V (2023). Advancing glaucoma detection with convolutional neural networks: a paradigm shift in ophthalmology. Rom J Ophthalmol.

[18] Nevska AO, Pohosian OA, Goncharuk KO, Sofyna DF, Chernenko OO, Tronko KM, Kozhan NI, Korol AR (2024). Detecting diabetic retinopathy using an artificial intelligence-based software platform: a pilot study. J. Ophthalmol. (Ukraine).

[19] Khramenko NI, Velychko LM, Konovalova NV (2023). Features of hemodynamic and immunological parameters in patients with recurrent uveitis complicated by hypertension, Fuchs heterochromic uveitis and Posner-Schlossman syndrome. Rom J Ophthalmol.

[20] Huang X, Islam MR, Akter S, Ahmed F, Kazami E, Serhan HA, Abd-Alrazaq A, Yousefi S (2023). Artificial intelligence in glaucoma: opportunities, challenges, and future directions. Biomed Eng Online.

[21] Delsoz M, Raja H, Madadi Y, Tang AA, Wirostko BM, Kahook MY, Yousefi S (2023). The Use of ChatGPT to Assist in Diagnosing Glaucoma Based on Clinical Case Reports. Ophthalmol Ther.

[22] Hirosawa T, Harada Y, Tokumasu K, Ito T, Suzuki T, Shimizu T (2024). Evaluating ChatGPT-4’s Diagnostic Accuracy: Impact of Visual Data Integration. JMIR Med Inform.

[23] Christopher M, Gonzalez R, Huynh J, Walker E, Radha Saseendrakumar B, Bowd C, Belghith A, Goldbaum MH, Fazio MA, Girkin CA, De Moraes CG, Liebmann JM, Weinreb RN, Baxter SL, Zangwill LM (2024). Proactive Decision Support for Glaucoma Treatment: Predicting Surgical Interventions with Clinically Available Data. Bioengineering (Basel).

